# The Gynæcological Fad

**Published:** 1895-07

**Authors:** 


### THE GYNÆCOLOGICAL FAD.

From the point of view of an observer who is not a gynæcologist, it appears that gynæcology has a Benjamin's share in current medical literature and a somewhat disproportional amount of consideration paid it.

The pages of our exchanges are largely filled with articles on subjects in this province and there is scarcely a town in the country so small that it does not boast of a specialist in diseases of women, with the usual complement of cœliotomies and the *furor operandi* strong upon him. Conservatism, meaning patience, is pushed to the wall by the demand for rapid results. Now, surgical gynæcology is mere tinkering, is the refrain of the chorus, and every little frog in the wayside pond adds his shrill pipe to the sonorous bellowings of his larger brothers. Aristophanes outdone!

Running through all this mass of excellent, bad and worse literature, like the red strand in the ropes of the British navy, is the pathological role of the gonococcus. The gonococcus is king of terrors! Death lurks in the diseased urethra and the mirth of the bride that "paced into the hall" such a short time ago, is changed to mourning. The ovaries are considered more in their pathological relation and bearing upon the theory of Malthus, than as organs essential to the propagation of the species and the maintenance of the human family.

The inference to be drawn from all this bustle and activity is that gynæcology is going through a period of evolution and that it means the building of the superstructure that will be solid and scientific and symmetrical some day, though not yet. And the apology for it is that each element must be separately tested before it goes into the formation of the complex whole. If, in the process of elaborating the art, human lives must be sacrificed, the sacrifice must be regarded from the broad platform of the general good that is to follow through experience, and not from the narrow standpoint of the desolated home. But such sacrifices are happily becoming less frequent with growth of knowledge and it may be prophesied with confidence that the "new woman" of the future will have a firmer grasp on her ovaries than had the "old woman of the past."

